# Genomic Amplification and Ovary-Specific Expression of Zona Pellucida Genes in Channel Catfish and Blue Catfish

**DOI:** 10.3390/ijms27209100

**Published:** 2026-10-14

**Authors:** Tong Chen, Dongya Gao, Baofeng Su, Rex Dunham, Zhanjiang Liu

**Affiliations:** 1 Department of Biology, College of Arts and Sciences, Tennessee Technological University, Cookeville, TN 38505, USA; tchen43@tntech.edu (T.C.); dgao@tntech.edu (D.G.); 2 Environmental Sciences Ph.D. Program, School of Environmental Studies, Box 5152, Tennessee Technological University, Cookeville, TN 38505, USA; 3 School of Fisheries, Aquaculture and Aquatic Sciences, Auburn University, Auburn, AL 36849, USA; bzs0014@auburn.edu (B.S.); dunhara@auburn.edu (R.D.)

**Keywords:** zona pellucida, sex, ovary differentiation, RNA-Seq, catfish

## Abstract

Channel catfish (*Ictalurus punctatus*) is the most important aquaculture species in the United States, with the interspecific hybrid made from mating female channel catfish with male blue catfish (*I. furcatus*) being the predominant genotype for the catfish industry. While studying the mechanisms of their reproductive isolation, we determined zona pellucida (ZP) genes in both species. While ZP genes are well known for their ovary-specific expression in higher vertebrates such as mammals, the site of their expression in fish varies depending on species. In this study, we annotated the ZP genes in channel catfish and blue catfish and conducted phylogenetic analysis. We found that ZP genes are highly amplified in the catfish genomes, with ZP3- and ZP4-like genes being the most abundant in the genomes, derived from tandem and segmental duplications. A total of 39 and 49 ZP genes (including 4 and 5 pseudogenes) in the blue catfish genome and channel catfish genome were identified, respectively. Expression analysis using RNA-Seq revealed ovary-specific expression in females, with no expression in the gonadal tissues of males, nor in any other tissues of either males or females. RT-PCR analysis indicated that the ZP genes were not expressed until 90 days post fertilization, consistent with the timing of oogenesis of the developing gonad. Single-nucleus RNA-seq (snRNA-Seq) confirmed expression of ZP genes only in oocytes. Future studies are warranted to determine the molecular mechanisms of sex-, tissue-, and time-specific expression of ZP genes, especially in relation to various taxa within teleost fish.

## 1. Introduction

Zona pellucida (ZP) domain-containing proteins are extracellular proteins surrounding oocytes of various organisms, ranging from *Caenorhabditis elegans* to mammals [1,2,3,4]. This layer of ZP proteins is referred to as zona pellucida in mammals, vitelline membrane in birds, vitelline envelope in amphibians, and chorion in fish [5]. There are three ZP genes in mouse [6], four in human [7], seven in chicken [8], and eleven in African clawed frog (*Xenopus laevis*) [9]. In teleost fish, the number of ZP genes varies, but are generally larger as compared to amphibians, birds, or mammals.

The nomenclature of ZP genes has evolved over time. Early studies in mouse used numerical designations of ZP1, ZP2, and ZP3 [6]. Later, a letter-based nomenclature was introduced, in which ZP2 was designated as ZPA because it had the highest number of amino acids, while ZP1 and ZP3 were designated ZPB and ZPC, respectively [10]. Such nomenclatures have led to considerable inconsistency among studies. Based on phylogenetic analysis, the most recent nomenclature classified ZP genes into six subfamilies, ZP1, ZP2/ZPA, ZP3/ZPC, ZP4/ZPB, ZPAX, and ZPD [11], which was followed in this paper.

ZP glycoproteins are expressed in the ovaries in mammalian species, but expression in both the liver and ovary tissues was reported in teleost fish: the switch in expression organs of ZP genes from the ovary to the liver was suggested to have occurred during the evolution of teleost [5]. For example, ZP genes of Japanese eel *Anguilla japonica* (Anguilliformes in Elopomorpha) are expressed in their ovaries; however, those of euteleostean fishes are expressed also in the liver of mature females and transported to the ovary [12,13,14,15,16]. These observations made Sano et al. hypothesize that ZP genes acquired the ability to be expressed in the liver in the common ancestor of Clupeocephala [5]. However, a consistent site of expression of ZP genes has not been validated. In a recent study, Zhang et al. reported that ZP genes were primarily expressed in the ovaries but expression was also observed in various other tissues, as well as in the testis of male fish of the Chinese tongue sole (*Cynoglossus semilaevis*); in particular, three genes, Cszpb2b, Cszpb2c and Cszpc2, were expressed at high levels in the liver [17].

Channel catfish (*Ictalurus punctatus*) and blue catfish (*I. furcatus*) are important for aquaculture in the United States. Although channel catfish is the primary cultured species, F1 hybrids made from mating channel catfish female and blue catfish male are now the predominant genotype in the US catfish industry. Although much is known about sex determination in both channel catfish and blue catfish [18,19,20,21], gonadal development and differentiation have not been studied. We identified sex-linked markers in channel catfish [22] and blue catfish [23]. Such sex markers allowed for the analysis of gene expression during early gonadal development in genetic males and females. The objectives of this study were as follows: (1) to annotate zona pellucida genes in the genomes of channel catfish and blue catfish; (2) to characterize their genomic structure, location, and organization; and (3) to determine their expression patterns in relation to sex, tissue, cell types, and time after fertilization. Here, we report genomic amplification of ZP3- and ZP4-like genes in the catfish genomes and their specific expression in the female gonad within oocyte cells.

## 2. Results

### 2.1. Zona Pellucida Genes in Catfish and Their Annotation

**Figure 1:**
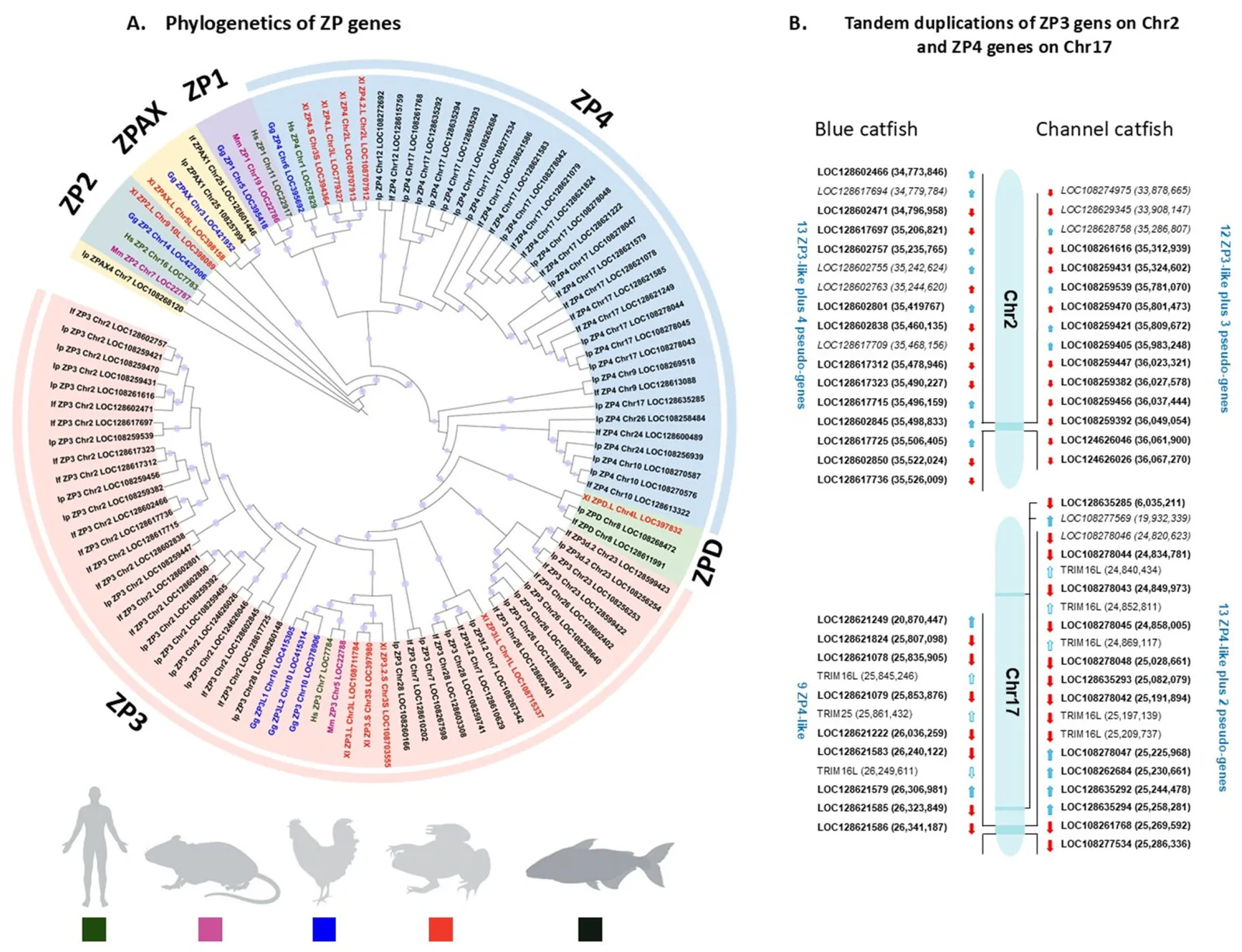
Phylogenetic analysis and amplification of ZP genes on chromosome 2 and chromosome 17. (**A**) A phylogenetic tree of ZP genes, assigning them to ZP1 (purple), ZP2 (light blue-gray), ZP3 (pink), ZP4 (blue), ZPAX (yellow), and ZPD (green) genes. Note that ZP1 and ZP2 are absent from catfish. The majority of ZP genes in catfish are ZP3 and ZP4 genes. ZP genes from selected species of major evolutionary groups of mammals (human and mouse), birds (chicken), amphibians (African clawed frog), and teleost fish [channel catfish (*Ictalurus punctatus*) and blue catfish (*I. furcatus*)] were used for phylogenetic analysis. Significant bootstrap values (>70%) are indicated by blue circles. (**B**) A schematic presentation of chromosomal locations and orientations (as indicated by the direction of the arrows) of ZP3 and ZP4 genes on chromosome 2 and chromosome 17 in the genomes of channel catfish and blue catfish. For channel catfish, twelve ZP3 plus three pseudogenes were annotated in chromosome 2. For blue catfish, thirteen ZP3 plus four pseudogenes were annotated in chromosome 2. In chromosome 17, thirteen ZP4 plus two pseudogenes and nine ZP4 genes are annotated in channel catfish and blue catfish, respectively. The red color indicates the presence of ZP genes on the forward strand as in the reference genome sequence, while blue indicates the presence of ZP genes on the complementary strand of the reference genome sequence. The orientation of ZP genes is indicated by the directions of the arrows. Non-shaded arrows indicate other genes intervening with the ZP genes in the genome neighborhood. The numbers in parentheses indicate genomic location in base pairs from the beginning of the reference genome sequence. Pseudogenes are italicized and not in bold whereas protein-coding ZP genes are in bold font.

Accurate gene annotation is essential for reliable analysis of gene expression and function. The nomenclature of ZP genes in mammals remains complex and inconsistent [24]. In addition, ZP genes are rapidly evolving genes, making their annotation challenging. To address this, we performed phylogenetic analyses to accurately annotate ZP genes in the genomes of blue catfish and channel catfish. These refined annotations provide a critical foundation for subsequent gene expression analysis. As shown in Figure 1A, six types of ZP genes exist among various vertebrate species, including ZP1, ZP2, ZP3, ZP4, ZPAX, and ZPD. However, not all six ZP genes exist in every major phylogenetic group. ZP1 is present only in reptiles, birds and mammals, but not in lower vertebrates including shark and lamprey, teleost fish and amphibians. ZP2 is present in almost each of the major vertebrate groups, but not necessarily in each species within each group. For instance, among teleost fish, ZP2 genes are present in rainbow trout and carp, but absent from channel catfish and blue catfish. A similar situation is observed with the ZPAX gene (Table 1). ZP3 and ZP4 genes are present in all vertebrate species under study, while ZPD genes are present in all teleost species examined, but absent from lampreys, lungfish, reptiles, birds and mammals (Table 1).

Annotation of ZP genes in blue catfish and channel catfish is summarized in Table 2 and Table 3, respectively. In blue catfish, one ZPAX gene was identified (LOC128601446) and one ZPD gene was identified (LOC128611991), but large numbers of ZP3 and ZP4 genes were identified (Table 2). Similar to the situation in blue catfish, only two ZPAX genes and one ZPD gene were identified from channel catfish, but large numbers of ZP3 and ZP4 genes were identified (Table 3).

**Table 2:** Zona pellucida genes in the genome of blue catfish (*I. furcatus*).

Locus	Gene Description	Strand	Chromosome and Position	Expression (FPKM)	Relative Expression
**LOC128601446**	ZP-AX1	−	Chr25 (17,287,916–17,293,856)	396.3	611.5
**LOC128611991**	ZP glycoprotein d	−	Chr8 (21,909,990–21,916,451)	2425.3	1250.8
**LOC128602466**	ZP3-like	−	Chr2 (34,773,846–34,776,582)	44.0	5287.6
**LOC128602471**	ZP3-like	+	Chr2 (34,796,958–34,801,213)	1382.5	2571.3
**LOC128602755**	ZP3-like (pseudogene)	−	Chr2 (35,242,624–35,243,619)	0	0
**LOC128602757**	ZP3-like	+	Chr2 (35,235,765–35,238,297)	3198.4	3040.7
**LOC128602763**	ZP3-like (pseudogene)	−	Chr2 (35,244,620–35,246,804)	0	0
**LOC128602801**	ZP3-like	−	Chr2 (35,419,767–35,421,738)	4443.0	3394.4
**LOC128602838**	ZP3-like	+	Chr2 (35,460,135–35,462,033)	4728.5	3343.4
**LOC128602845**	ZP3-like	−	Chr2 (35,498,833–35,500,379)	8021.0	3359.4
**LOC128602850**	ZP3-like	+	Chr2 (35,522,024–35,525,259)	3778.9	6039.0
**LOC128617312**	ZP3-like	+	Chr2 (35,478,946–35,482,310)	3318.3	2884.6
**LOC128617323**	ZP3-like	+	Chr2 (35,490,227–35,492,964)	4436.5	4030.7
**LOC128617694**	ZP3-like (pseudogene)	−	Chr2 (34,779,784–34,781,743)	0	0
**LOC128617697**	ZP3-like	−	Chr2 (35,206,821–35,214,723)	139.1	2043.4
**LOC128617709**	ZP3-like (pseudogene)	+	Chr2 (35,468,156–35,470,084)	0	0
**LOC128617715**	ZP3-like	−	Chr2 (35,496,159–35,498,635)	231.3	26,984.0
**LOC128617725**	ZP3-like	−	Chr2 (35,506,405–35,515,504)	9232.2	3105.6
**LOC128617736**	ZP3-like	+	Chr2 (35,526,009–35,527,165)	116.8	12,618.4
**LOC128610629**	ZP glycoprotein 3f, tandem duplicate 2	+	Chr7 (240,949–245,921)	372.8	2018.8
**LOC128610202**	ZP3-like	−	Chr7 (23,956,329–23,963,205)	86.4	45.5
**LOC128599422**	ZP3-like	+	Chr23 (20,708,187–20,712,114)	194.1	226.2
**LOC128602402**	ZP3-like	+	Chr26 (10,346,333–10,348,329)	2490.9	2606.3
**LOC128602401**	ZP3-like	+	Chr26 (10,339,797–10,341,781)	3598.9	2879.3
**LOC128603308**	ZP3 isoform X2	−	Chr28 (15,154,633–15,157,764)	775.1	2669.8
**LOC128599423**	ZP3d.2 isoform X5	+	Chr23 (20,715,346–20,720,923)	31.8	27.9
**LOC128613088**	ZP4-like	−	Chr9 (29,146,776–29,149,789)	2556.7	4637.5
**LOC128613322**	ZP4-like	−	Chr10 (26,640,129–26,645,601)	438.3	1323.5
**LOC128615759**	ZP4-like	−	Chr12 (9,713,376–9,716,483)	242.7	283.2
**LOC128600489**	ZP4-like	−	Chr24 (18,819,875–18,821,086)	505.3	486.3
**LOC128621579**	ZP4-like	−	Chr17 (26,306,981–26,308,874)	13,902.5	2402.7
**LOC128621249**	ZP4-like	−	Chr17 (20,870,447–20,872,815)	3427.1	1907.9
**LOC128621824**	ZP4-like	+	Chr17 (25,807,098–25,810,768)	2883.6	2985.3
**LOC128621078**	ZP4-like	+	Chr17 (25,835,905–25,837,370)	1470.4	28,802.2
**LOC128621079**	ZP4-like	+	Chr17 (25,853,876–25,855,797)	8006.1	4497.2
**LOC128621222**	ZP4-like	+	Chr17 (26,036,259–26,039,134)	10,038.7	4221.7
**LOC128621583**	ZP4-like	+	Chr17 (26,240,122–26,242,044)	21,045.5	3737.5
**LOC128621585**	ZP4-like	+	Chr17 (26,323,894–26,325,847)	35,146.8	3053.1
**LOC128621586**	ZP4-like	+	Chr17 (26,341,187–26,343,068)	38,590.3	3289.2

**Figure 2:**
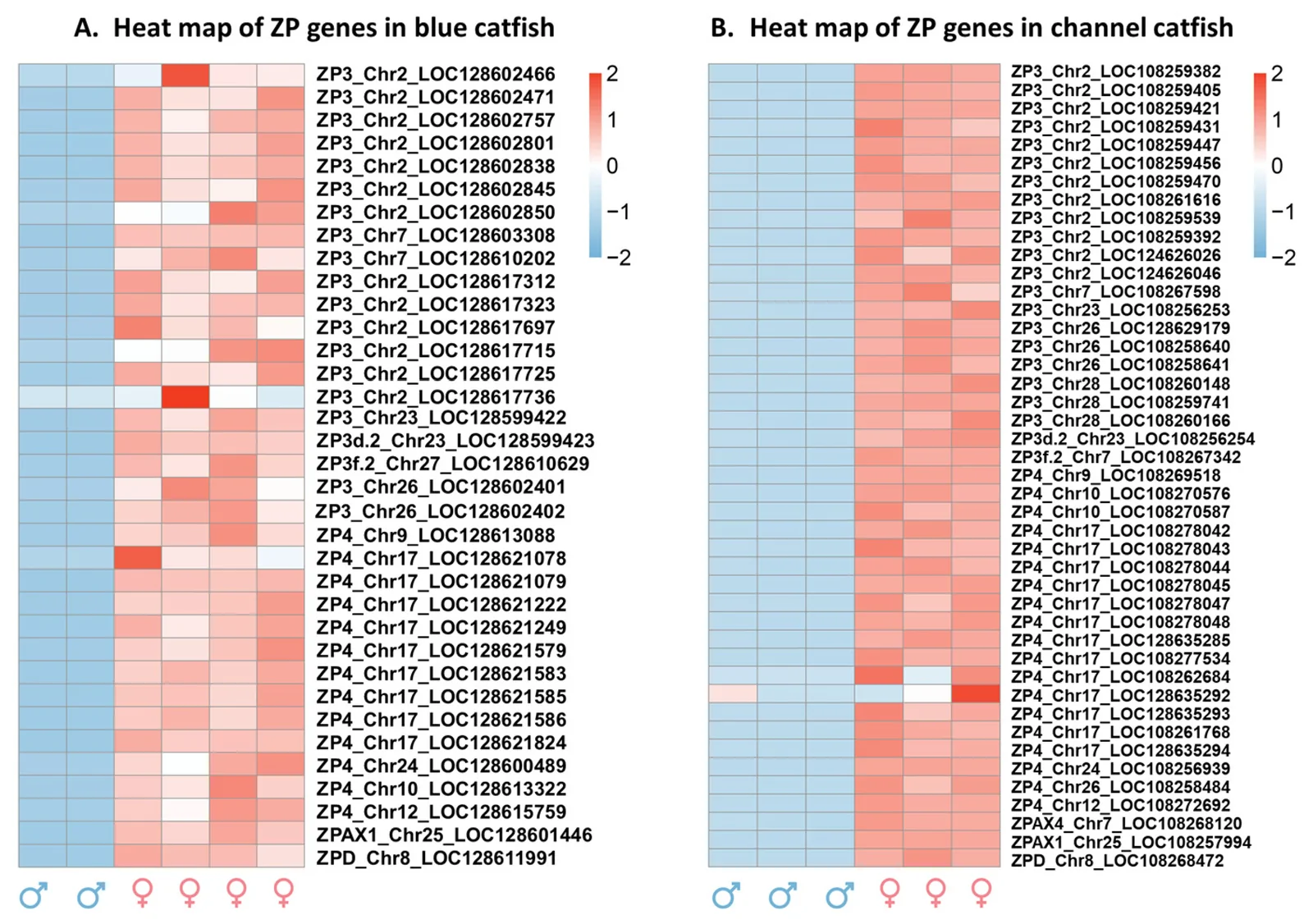
Heatmaps of expresssion of ZP genes in the gonad tissue of blue catfish (**A**) and channel catfish (**B**). Expression levels of ZP genes were quantified as fragment per kilobase per million (FPKM) based on RNA-Seq data from gonadal tissues of female or male blue catfish and channel catfish at 135 days post fertilization. Gene expression levels are color-coded from low (blue) to high (red). Each row represents one gene, with their identities listed on the right.

ZP3 genes are shaded in pink and ZP4 genes are shaded in blue. ZP3 genes are mostly located on chromosome 2 but those on other chromosomes are shaded in dark blue. Similarly, ZP4 genes are mostly located on chromosome 17 but those on other chromosomes are shaded in dark pink. ZPAX and ZPD genes are shaded in gray. “+” strand is the same strand as the reference genome sequence and “−” strand is the complementary strand. FPKM represents fragment per kilobase of transcript per million mapped reads of RNA-Seq data from gonad tissues of female blue catfish at 135 days post fertilization. Relative expression is the fold expression in the female compared to in the male. Expression in females is drastically higher than in males, reflecting that RPKM from males is largely due to the very high sensitivity of RNA-Seq. The background color in the rows of the table indicates each of the four categories of ZP genes, ZPAX, ZP3, ZP4, and ZPD. Expression (FPKM) is derived from RNA-Seq datasets using the average value of FPKM of individuals, as shown in Figure 2A.

**Table 3:** ZP genes in channel catfish (*I. punctatus*).

Locus	Gene Description	Strand	Chromosome and Position	Expression (FPKM)	Relative Expression
**LOC108257994**	ZP protein AX1	−	Chr25 (16,546,991–16,556,749)	550.0	916.4
**LOC108268120**	ZP protein AX4	−	Chr7 (18,149,030–18,159,139)	33.3	31.5
**LOC108268472**	ZP glycoprotein d	−	Chr8 (21,557,568–21,562,724)	2198.2	815.1
**LOC108274975**	ZP 3-like (pseudogene)	+	Chr2 (33,878,665–33,885,252)	0	0
**LOC128629345**	ZP 3-like (pseudogene)	+	Chr2 (33,908,147–33,908,957)	0	0
**LOC128628758**	ZP 3-like (pseudogene)	−	Chr2 (35,286,807–35,289,938)	0	0
**LOC108261616**	ZP3	+	Chr2 (35,312,939–35,314,977)	860.8	965.6
**LOC108259431**	ZP3	+	Chr2 (35,324,602–35,326,634)	1041.4	1777.0
**LOC108259539**	ZP3	−	Chr2 (35,781,070–35,783,096)	40.6	289.8
**LOC108259470**	ZP3	+	Chr2 (35,801,473–35,803,428)	2148.4	789.9
**LOC108259421**	ZP 3-like	−	Chr2 (35,809,672–35,811,739)	2251.9	2911.2
**LOC108259405**	ZP3	−	Chr2 (35,983,248–36,074,164)	2619.9	997.4
**LOC108259447**	ZP3	+	Chr2 (36,023,321–36,025,351)	2573.5	943.1
**LOC108259382**	ZP3	+	Chr2 (36,027,578–36,029,610)	1787.2	1392.1
**LOC108259456**	ZP3	+	Chr2 (36,037,444–36,039,448)	2050.5	617.1
**LOC108259392**	ZP3	+	Chr2 (36,049,054–36,086,916)	2429.7	1001.0
**LOC124626046**	ZP3	+	Chr2 (36,061,900–36,063,911)	145.3	205.3
**LOC124626026**	ZP3	+	Chr2 (36,067,270–36,069,285)	882.9	2447.4
**LOC108267598**	ZP3	−	Chr7 (22,566,636–22,574,389)	30.8	22.6
**LOC108267342**	ZP glycoprotein 3f tandem duplicate 2	+	Chr7 (130,320–133,187)	1494.0	749.6
**LOC108256253**	ZP3	+	Chr23 (20,309,165–20,314,327)	243.0	284.3
**LOC108256254**	ZP3d.2 isoform X5	+	Chr23 (20,315,818–20,321,501)	206.2	109.7
**LOC108258641**	ZP3	+	Chr26 (10,671,101–10,673,716)	908.9	3953.9
**LOC128629179**	ZP3	+	Chr26 (10,681,044–10,683,672)	895.6	2908.7
**LOC108258640**	ZP3	+	Chr26 (10,687,256–10,689,841)	1966.6	2807.8
**LOC108260166**	ZP3	−	Chr28 (20,008,919–20,013,117)	121.7	88.8
**LOC108260148**	ZP3	+	Chr28 (20,062,660–20,066,978)	110.4	183.6
**LOC108259741**	ZP3 isoform X2	−	Chr28 (15,258,224–15,367,957)	1383.1	2690.7
**LOC108269518**	ZP4	−	Chr9 (29,595,148–29,598,449)	2656.9	2554.8
**LOC108270587**	ZP4	+	Chr10 (22,350,060–22,353,539)	1472.5	1663.1
**LOC108270576**	ZP4-like	−	Chr10 (25,684,144–25,687,758)	1228.6	1059.0
**LOC108272692**	ZP4	−	Chr12 (13,397,888–13,401,013)	231.9	247.7
**LOC108256939**	ZP4	−	Chr24 (14,760,712–14,764,041)	2591.9	2710.0
**LOC108258484**	ZP4-like isoform X2	+	Chr26 (13,669,209–13,678,880)	321.0	231.7
**LOC108277569**	ZP4-like (pseudogene)	−	Chr17 (19,932,339–19,935,766)	0	0
**LOC108278046**	ZP4-like (pseudogene)	+	Chr17 (24,820,623–24,822,445)	0	0
**LOC108278044**	ZP4	+	Chr17 (24,834,781–24,836,678)	838.7	581.1
**LOC108278043**	ZP4	+	Chr17 (24,849,973–24,851,839)	1151.3	666.5
**LOC108278045**	ZP4-like	+	Chr17 (24,858,005–24,859,886)	1770.6	668.9
**LOC108278048**	ZP4	+	Chr17 (25,028,661–25,030,471)	3536.6	894.7
**LOC128635293**	ZP4-like	+	Chr17 (25,082,079–25,263,597)	52.6	1020.4
**LOC108278042**	ZP4	+	Chr17 (25,191,894–25,193,783)	2564.8	1130.7
**LOC108278047**	ZP4	−	Chr17 (25,225,968–25,227,827)	1471.6	468.9
**LOC108262684**	ZP4-like	−	Chr17 (25,230,661–25,232,531)	5.5	583.9
**LOC128635292**	ZP4-like	−	Chr17 (25,244,478–25,246,348)	0.2	4.9
**LOC128635294**	ZP4-like	−	Chr17 (25,258,281–25,260,152)	50.2	369.0
**LOC108261768**	ZP4	+	Chr17 (25,269,592–25,271,455)	898.0	1250.3
**LOC108277534**	ZP4	+	Chr17 (25,286,336–25,288,205)	2101.8	820.6
**LOC128635285**	ZP4	+	Chr17 (6,035,211–6,041,602)	61.0	117.9

ZP3 and ZP4 genes are shaded in green and blue, respectively. ZP3 genes are mostly located on chromosome 2 but those on other chromosomes are shaded in dark blue. Similarly, ZP4 genes are mostly located on chromosome 17 but those on other chromosomes are shaded in dark green. ZPAX and ZPD genes are shaded in gray. “+” strand is the same strand as the reference genome sequence and “−” strand is the complementary strand. FPKM represents fragment per kilobase per million of RNA-Seq data of the gonad channel. Relative expression is the fold expression in the female compared to in the male. Again, low numbers of FPKM in the male reflect the sensitivity of RNA-Seq analysis. Expression levels in FPKM were derived from RNA-Seq datasets using the average value of FPKM of individuals, as shown in Figure 2B.

### 2.2. ZP3 and ZP4 Genes Are Amplified in Catfish Genomes

ZP3 and ZP4 genes have high numbers of copies in the genomes of blue catfish and channel catfish, with the blue catfish genome harboring 20 ZP3 genes and 13 ZP4 genes (Table 2) and the channel catfish genome harboring 22 ZP3 genes and 19 ZP4 genes. In addition to these, four and five pseudogenes are present in the blue catfish genome and channel catfish genome, respectively. The proximity of the genomic locations of the identified ZP3 and ZP4 genes in blue catfish and channel catfish suggests that they were from both tandem duplications and fragmental duplications (Figure 1B). In addition to the high level of gene duplications on chromosome 2 and chromosome 17, gene duplications of both ZP3 and ZP4 were also observed on chromosomes 7, 23 and 26 in blue catfish and on chromosomes 7, 10, 23, 26 and 28 in channel catfish (Table 2 and Table 3).

### 2.3. Zona Pellucida Genes Are Expressed Only in Females

Zona pellucida (ZP) genes exhibit female-specific expression patterns. Comparative transcriptome analysis demonstrated consistent female-specific expression of ZP genes in both blue catfish and channel catfish. In blue catfish, 20 ZP3 genes, 13 ZP4 genes, along with ZPAX1 and ZPD, were exclusively expressed in female gonadal tissues at 135 days post fertilization (Figure 2A, Table 2). Similarly, in channel catfish, 22 ZP3 genes, 19 ZP4 genes, together with ZPAX1, ZPAX4, and ZPD, exhibited female-specific expression (Figure 2B, Table 3). Only very low expression was noticed with a ZP4-like gene (LOC128635292) in channel catfish, but as shown in Table 3, this gene was expressed at low levels, even in the gonad of females, making it more prominent on the heatmap (Figure 2B) than absolute levels of expression.

### 2.4. Zona Pellucida Genes Are Only Expressed in the Ovaries of Catfish

**Figure 3:**
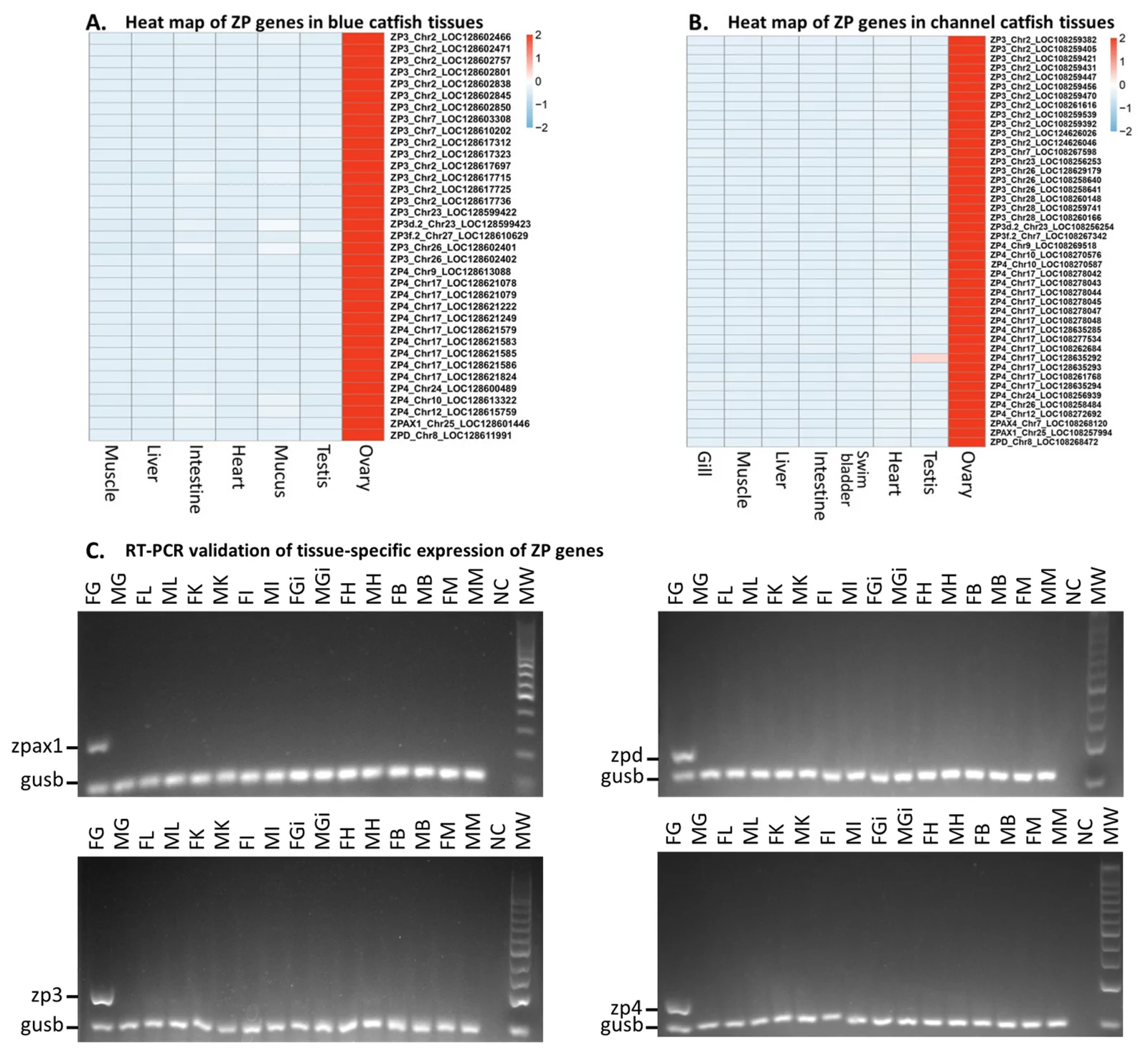
Gonad-specific expression of ZP genes in blue catfish (**A**) and channel catfish (**B**). Expression levels of ZP genes were quantified as fragment per kilobase of transcript per million mapped reads (FPKM) based on RNA-Seq data. Gene expression levels are color-coded from low (blue) to high (red). Each row represents one gene (listed on the right). RNA-Seq datasets were from previous projects (see Section 4). Note that for the heatmap of both blue catfish (**A**) and channel catfish (**B**), ovary and testis tissues were collected at 135 days post fertilization (dpf), while time of sample collection for other tissues varied: muscle, liver, intestine, heart and mucus tissues of blue catfish (**A**) were collected at 10.8 months of age, whereas the samples of channel catfish were collected from fish of approximately four months old, as detailed in Section 4. Nonetheless, ZP genes were expressed only in the ovary, not in the testis, nor in any other tissues regardless of the sex or age of the fish. (**C**) RT-PCR analysis of expression of four ZP genes (ZPAX1, ZPD, ZP3, ZP4) in different tissues of male and female blue catfish at 135 dpf. FG—female gonad; MG—male gonad; FL—female liver; ML—male liver; FK—female kidney; MK—male kidney; FGi—female gill; MGi—male gill; FH—female heart; MH—male heart; FB—female brain; MB—male brain; FM—female muscle; MM—male muscle. The beta-glucuronidase (gusb) gene was used as an internal positive control [23]. Note again that the tested ZP genes (ZPAX1, ZPD, ZP3 and ZP4) were expressed only in the ovary, but not in the testis, nor in any other tissues of male or female fish.

In mammalian species, ZP genes are expressed exclusively in the ovaries, whereas among teleost fish, their expression has been reported in both the liver and the ovary in some species. To deal with this large number of ZP genes in both channel catfish and blue catfish, RNA-Seq analysis was used. Using RNA-Seq datasets, we determined expression of the ZP genes in various tissues. As shown in Figure 3, all ZP genes are exclusively expressed in ovary tissues, but not in any other tissues, including the muscle, liver, intestine, heart, and testis in both channel catfish and blue catfish and the swim bladder of channel catfish. For blue catfish, there is no expression in any other tissues but the ovary (Figure 3A). For channel catfish, the only exception is low expression of a ZP4-like gene (LOC128635292) in the testis tissue at 135 days post fertilization (Figure 3B). However, as discussed above, this gene was expressed at low levels in both female and male gonadal tissues (Table 3). In addition, channel catfish RNA-Seq analysis was conducted using pooled samples after identification of genetic sex [31]; however, it is possible that one individual in the mixed pool of the “male” could have been a genetic female, as determined by PCR using sex-linked markers, that might have had a sex reversal [19,22]. Blue catfish RNA-Seq analysis was conducted with individual fish, and therefore, there was no possibility of mixture of tissues, providing strong evidence for sex-specific expression of ZP genes only in females, as shown in Figure 2. With large numbers of genes and so many tissues, we did not attempt to validate ovary-specific expression for all of these genes but instead randomly selected one ZP gene from each of four subfamilies: ZPAX1 (LOC128601446), ZPD (LOC128611991), ZP3 (LOC128602850) and ZP4 (LOC128621078). As shown in Figure 3C, RT-PCR analysis confirmed female-specific expression and ovary-specific expression (Figure 3C).

### 2.5. ZP Genes Are Exclusively Expressed Within Oocytes

**Figure 4:**
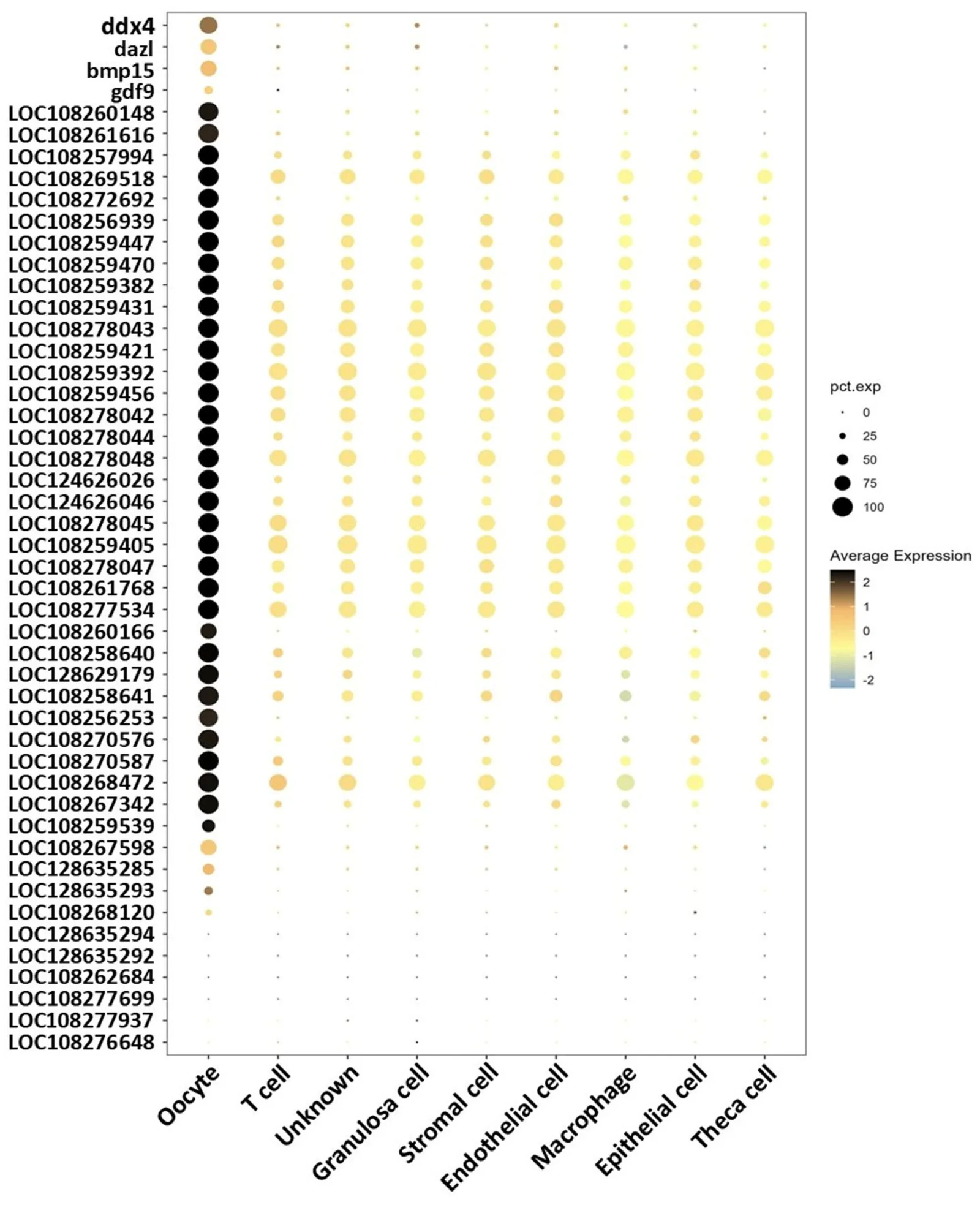
Analysis of expression of ZP genes in the ovaries using single-nucleus RNA-Seq (snRNA-Seq). snRNA-Seq was conducted using female gonad tissues of channel catfish. Genes expressed in single cells were clustered and annotated. We used ddx4, dazl, gdf9 and bmp15 as markers for oocytes [11,32,33]. Of the 44 ZP genes in channel catfish, 37 showed detectable expression in the snRNA-seq dataset, whereas 7 genes (LOC108268120, LOC128635294, LOC128635292, LOC108262684, LOC108277699, LOC108277937, and LOC108276648) showed very low expression across the annotated cell populations.

To determine in what cell types ZP genes were expressed, single-nucleus RNA-Seq (snRNA-Seq) was conducted. Data of snRNA-Seq have been deposited in the NCBI. As shown in Figure 4, ZP genes were exclusively expressed in oocytes, but not in other cell types such as T cells, follicle cells, granulosa cells, stromal cells, endothelial cells, microphages, epithelial cells, and theca cells within ovary tissues.

### 2.6. Temporal Expression of Zona Pellucida Genes

**Figure 5:**
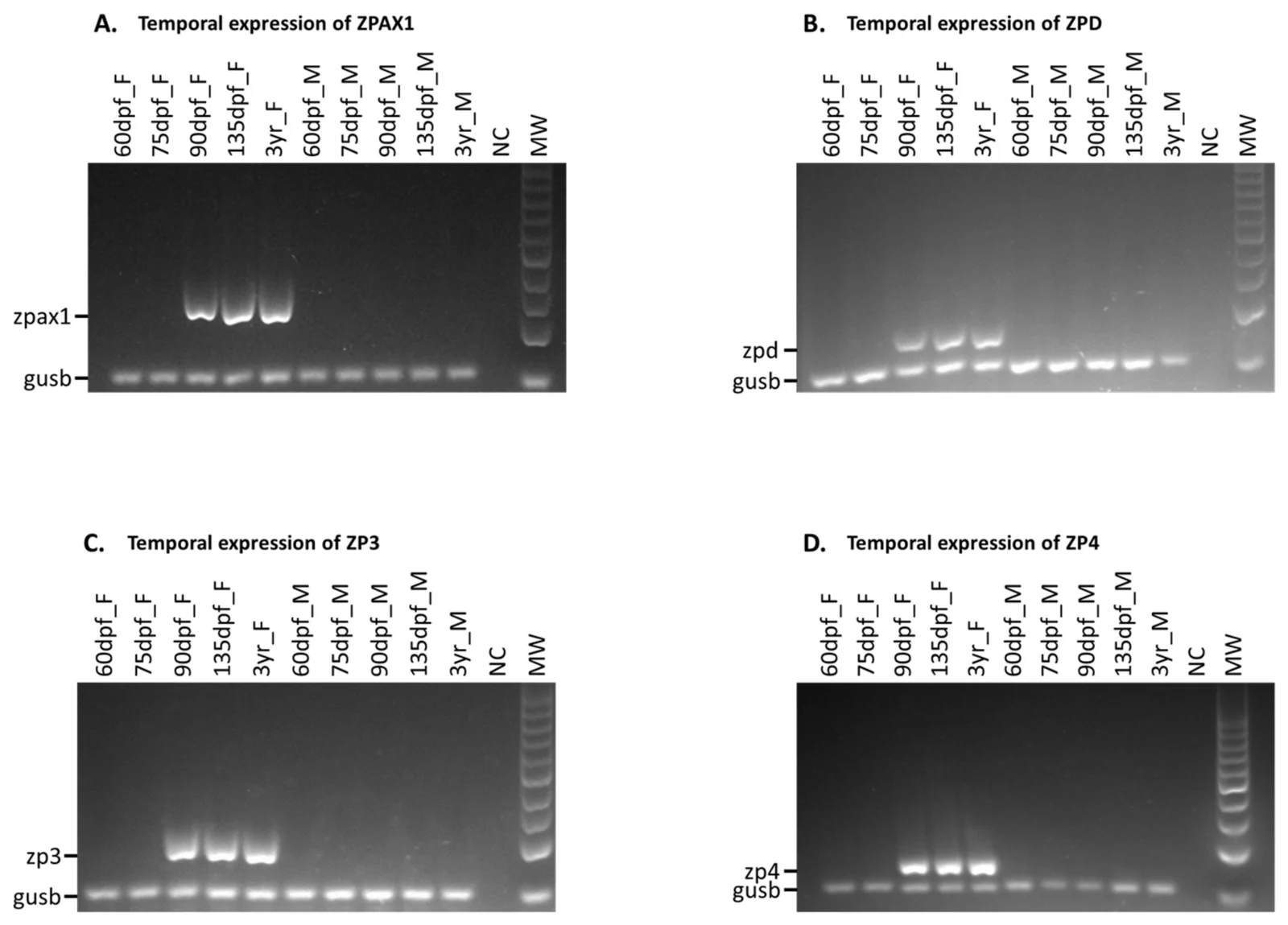
Temporal expression patterns of four ZP genes analyzed by RT-PCR. RT-PCR analysis of ZPAX1 (**A**), ZPD (**B**), ZP3 (**C**), and ZP4 (**D**) in gonadal tissues of female (F) and male (M) blue catfish at 60, 75, 90, and 135 days post fertilization (dpf) and 3 years of age. Beta-glucuronidase (gusb) was used as an internal control. NC, negative control; MW, molecular weight marker.

To determine when ZP genes were expressed, female gonadal tissues of blue catfish were collected at various times after fertilization. The same four ZP genes, as discussed above, were used as representatives of the ZP gene subfamily. As shown in Figure 5, the four ZP genes were not expressed before 90 days after fertilization. Expression was detected in the female gonad tissues at 90 days, 135 days, and in mature fish, but was not detected in the female gonad tissues at 60 and 75 days after fertilization. This is consistent with our observed timing of oogenesis within the developing gonad tissues, but detailed research is lacking.

## 3. Discussion

In this work, we annotated ZP genes in the genomes of channel catfish and blue catfish and characterized their genomic structure, organization, and expression. We report here massive gene amplification of ZP3-like and ZP4-like genes on chromosome 2 and chromosome 17, and female- and ovary-specific expression of ZP genes. We also conducted meta-analysis using all relevant existing RNA-Seq datasets of channel catfish and blue catfish and demonstrated exclusive expression of ZP genes only in the ovary, and only within oocytes, with expression detectable at 90 days post fertilization (dpf) or later, but not at 60 dpf and 75 dpf. This temporal and spatial expression indicated highly regulated expression of ZP genes in the growing oocytes.

**Figure 6:**
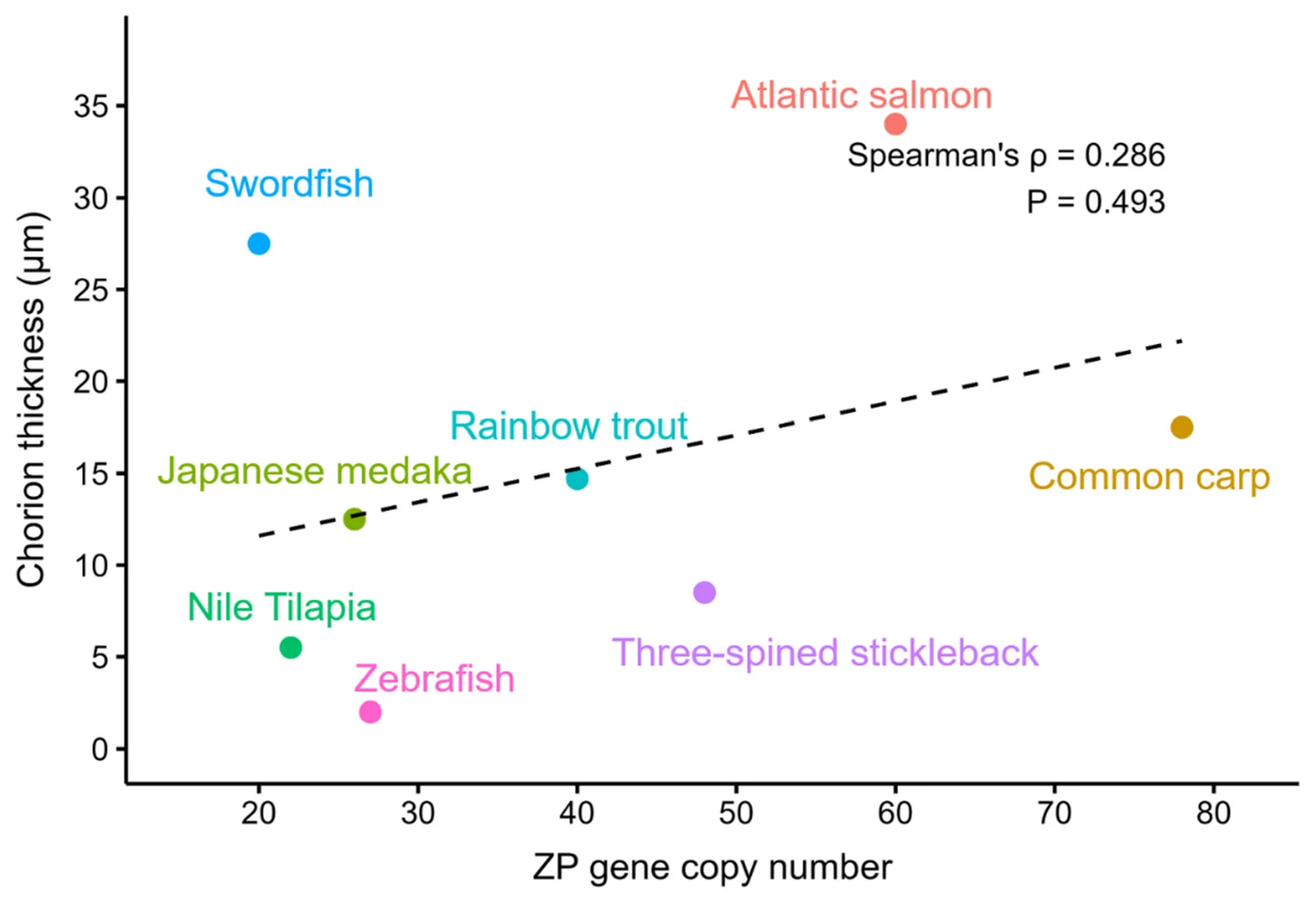
Determination of the correlation of ZP gene copy numbers with the thickness of chorion layers of various fish species for which such data are available (see Table 1). Spearman’s rank correlation test was conducted using *cor* function in R (version 4.3.1) to determine the relationship between the thickness of chorion layers and the copy numbers of ZP genes. Genome copy numbers across various species are provided in Supplementary Table S1. We note a weak positive correlation (ρ = 0.286), but it is not statistically significant (*p* = 0.493).

Massive amplifications of ZP3-like and ZP4-like genes were found in both channel catfish and blue catfish, while only a single copy of ZPAX (except channel catfish with two copies of ZPAX) and ZPD was found in the genomes of catfish. Channel catfish carry 20 to 22 copies of *ZP3*-like genes and blue catfish carry 13 to 19 copies of *ZP4*-like genes. These are tandemly amplified across chromosomes 2 and 17, likely to maximize protein synthesis to meet the massive demands of chorion development within the ovary. While Sano et al. found a correlation between the emergence of liver-synthesized ZP proteins and the thickness of chorion layers [27], we attempted to determine if genome copy numbers are correlated with the thickness of chorion layers among teleost species (Table 3) and conducted statistical correlation analysis. As shown in Figure 6, a weak positive correlation was found, but was not statistically significant (Spearman’s ρ = 0.286, *p* = 0.493), suggesting that regulation may occur at various levels to meet the demands of ZP proteins. Although no studies have been conducted on the thickness of chorion layers in channel catfish and blue catfish, our experience in working with microinjection of their embryos indicated that their chorion layers are quite thick. Nonetheless, genomic amplification of ZP3- and ZP4-like genes in channel catfish and blue catfish is quite notable, suggesting that they could have been amplified because of the high demands of the structural components of their thick chorion layers. A trend was observed that higher taxa in the evolutionary spectrum harbor fewer ZP genes than lower taxa; this trend tended to be the case within various teleost groups, while the effect of whole-genome duplication in allotetraploids or pseudo-autotetraploids was considered.

ZP genes of both channel catfish and blue catfish are expressed only in the females, and only in the ovaries, not in the liver or any other tissues. This is consistent with our expectations because they are lower teleost fish belonging to Siluriformes within Ostariophysi (which includes Cypriniformes and Siluriformes). Our meta-analysis of multiple RNA-Seq datasets of both channel catfish and blue catfish demonstrated abundant expression of ZP genes in the gonad tissues of females but not in the gonad tissues of males, nor in any other analyzed tissues in either females or males. Single-nucleus RNA-Seq further demonstrated that ZP genes were expressed only in oocytes (Figure 4). Expression of ZP genes is known to be regulated by the female-biased expression of FIGLA which forms heterodimers that bind to upstream E-box cis-regulatory sequences [34]. Here, in channel catfish and blue catfish, expression of FIGLA genes in the gonad of females of channel catfish and blue catfish is drastically higher than that in the gonad of male fish (Supplementary Table S2), approximately over 30 times more in females than in males in the gonad tissues. Additionally, epigenetic modifications such as DNA methylation and spatial regulation through chromosomal architecture could also regulate ovary-specific expression of ZP genes. How sex- and ovary-specific expression is achieved and regulated in catfish requires further study.

In conclusion, we identified and annotated ZP genes in both blue catfish and channel catfish. In these catfish species, the majority of ZP genes are ZP3- and ZP4-related, while just a few genes are related to ZPAX and ZPD (Figure 1A). Genomic location, gene context and gene orientations indicated that the large numbers of ZP3 and ZP4 genes were from tandem or segmental duplications, mostly on chromosome 2 and chromosome 17. Although expression of ZP genes in various tissues, especially in the liver, was reported in several fish species, this study provided no evidence of ZP expression in any tissue other than the gonad tissue of females, specifically in oocytes, suggesting highly controlled tissue-specific expression of ZP genes in catfish.

## 4. Materials and Methods

### 4.1. Experimental Fish

All fish work was conducted at Auburn University under a protocol approved by the Auburn University Institutional Animal Care and Use Committee (IACUC). Artificial spawning, fertilization, and fish husbandry were conducted as previously described [35]. Fish fries were reared in 456 L troughs under standard culture conditions. Fish were sampled at 60, 75, 90, and 135 days post fertilization (dpf), as well as at 3 years of age, for analysis of expression of ZP genes at different times post fertilization.

### 4.2. Tissue Collection, Sex Identification, and RNA Isolation

Gonad, muscle, liver, heart, kidney, intestine, brain, and gill tissues were collected as specified for the relevant experiments. For juvenile fish, each gonad sample was divided into two portions: one portion was used for DNA extraction and genetic sex identification, and the other portion was used for RNA extraction and gene expression analysis. Genetic sex was determined using a previously validated sex-specific molecular marker [23]. Tissues used for RNA extraction were immediately flash-frozen in liquid nitrogen and stored at −80 °C until use. Total RNA was extracted using the Quick-DNA/RNA Microprep Plus Kit according to the manufacturer’s instructions (Zymo Research Corporation, Orange, CA, USA). RNA concentration and quality were assessed using a NanoDrop spectrophotometer (Thermo Scientific, Wilmington, DE, USA). mRNA was reverse-transcribed into cDNA using the qScript™ cDNA Synthesis Kit (QuantaBio, Beverly, MA, USA) according to the manufacturer’s instructions. The resulting cDNA samples were diluted to 200 ng/μL for subsequent RT-PCR analysis.

### 4.3. RT-PCR Analysis

The primers were designed by using the NCBI Primer designing tool. The primers used for RT-PCR are listed in Table 4. Beta-glucuronidase (gusb) was used as an internal reference gene [24]. PCR amplification was performed under the following conditions: initial denaturation at 95 °C for 3 min, followed by 34 cycles of 95 °C for 20 s, gene-specific annealing temperatures for 15 s, and 72 °C for 25 s, with a final extension at 72 °C for 4 min and a hold at 4 °C. The annealing temperature for each of the tested genes and the control is listed in Table 4. PCR products were analyzed by agarose gel electrophoresis.

### 4.4. Annotation of Zona Pellucida Genes in Catfish

The reference genomes of both channel catfish (Coco_2.0) and blue catfish (Billie_1.0) were already annotated at the NCBI [36,37]. To identify all ZP genes in the genomes, we first conduction a keyword search of the NCBI’s reference genome annotation network to pull out all ZP genes that have been annotated from channel catfish, blue catfish, and zebrafish genomes. These gene sequences were then used as queries for TBLASTN searches to generate a pool of sequences homologous to ZP genes. A set of cutoff values were used for the homologous sequences: E-value threshold of ≤1 × 10^−10^, bit score > 100, sequence identity ≥ 60%, and alignment length ≥ 70 amino acids. The obtained ZP gene sequences were first analyzed to eliminate pseudogenes, based on whether an open reading frame was present and whether the gene was transcribed at all. The ZP gene sequences were then subjected to phylogenetic analysis for their annotation.

### 4.5. Phylogenetic Analysis

Phylogenetic analyses were conducted using zona pellucida genes from humans (*Homo sapiens*), mouse (*Mus musculus*), chicken (*Gallus gallus*), African clawed frog (*Xenopus laevis*), and several fish species including channel catfish and blue catfish. For phylogenetic analysis, we used DNASTAR MegAlign Pro^®^ (version 19.0; DNASTAR, Inc., Madison, WI, USA). Protein sequences were first aligned using MUSCLE (version 3.8.31) implemented in DNASTAR MegAlign Pro. Phylogenetic reconstruction was then performed using IQ-TREE with the maximum-likelihood method under the VT+F+R8 substitution model with 1000 bootstrap replicates. The tree file was visualized using iTOL (version 7.2.1) [38]. Chromosomal locations of zona pellucida genes in the genomes of channel catfish and blue catfish were determined by examining the gene sequences within the annotated reference genomes.

### 4.6. RNA-Seq Analysis and RNA-Seq Datasets

Transcriptome datasets of gonadal tissues of channel catfish at 135 dpf were downloaded from the SRA database (BioProject accession numbers PRJNA837725). In addition, RNA-seq datasets from various tissues of channel catfish, including the liver and gill (NCBI Sequence Read Archive accession number SRA049427) [39], swim bladder (BioProject accession numbers PRJNA400910) [40], and intestine (NCBI Sequence Read Archive accession number SRP009069), were also used [41]. RNA-seq data of gonad tissue from 135 dpf blue catfish were obtained from our previously published work [42]. RNA-seq data from the muscle, liver, intestine, heart and mucus of 10.8-month-old blue catfish were obtained from BioProject PRJNA1040907 [43]. The raw data of blue catfish and channel catfish were filtered to remove low-quality reads and sequencing adapters using fastp (version 0.23.4) [44]. Clean reads were mapped to the reference genomes of channel catfish, Coco_2.0 (GCF_001660625.3), and of blue catfish, Billie_1.0 (GCF_023375685.1), using HISAT2 (version 2.2.1) [45]. Aligned data generated by HISAT2 were converted to BAM format and subsequently sorted using Samtools (version 1.17) [46]. Featurecount (version 2.0.6) was employed to estimate the expression abundance of transcripts [47]. The fragment per kilobase of transcript per million mapped reads (FPKM) was applied to quantify gene transcription levels. Differentially expressed genes (DEGs) were determined by DESeq2 (version 1.40.2) [48]. An adjusted *p*-value ≤ 0.05 and two-fold difference in expression levels between males and females were used as thresholds to identify DEGs between sexes. Patterns of ZP gene expression were visualized as a heatmap using the R package pheatmap (version 1.0.13).

### 4.7. Single-Nucleus RNA Sequencing (snRNA-seq) and Data Analysis

Female gonadal tissues were collected from channel catfish at 135 days post fertilization (dpf) and stored in liquid nitrogen. Single-nucleus RNA Sequencing (snRNA-Seq) was conducted by CD Genomics (Shirley, NY, USA). The snRNA library was generated using Chromium GEM-X Single Cell 3′ Reagent Kits v4, which deliver a scalable microfluidic platform for 3′ digital gene expression by profiling 500–20,000 individual cells per sample, with each of the cells partitioned into nanoliter-scale Gel Beads-in-emulsion (GEMs), where all generated cDNA (from poly-adenylated mRNAs) share a common 10x Barcode. snRNA-Seq raw data were filtered and quality-checked with fastp v0.20.0 [44]. The filtered reads were subsequently mapped to the reference genome of channel catfish, Coco_2.0 (GCF_001660625.3), Ref. [36] and counted using the CellRanger v9.0.0 function count to generate the unique molecular identifier (UMI) matrix with default parameters [49]. Downstream analysis of the gene count matrix generated by CellRanger was performed in R version 4.1.0 using Seurat v5.2.1 [50]. To further obtain high-quality data, we performed quality control to filter low-quality cells and genes. In brief, cells were prefiltered according to the expression of at least 100 genes in each cell and each gene expressed in at least 3 cells. In addition, cells were filtered out (excluded) based on the following criteria: nFeature_RNA > 100, nFeature_RNA < 10,000, and nCount_RNA < 20,000. The gene expression count data were then normalized using the ‘NormalizeData’ function and scaled using the ‘ScaleData’ function, and we identified the top 2000 high-variable features using the ‘FindVariableFeatures’ function. “RunPCA” was performed to identify significantly principal components. The cells were clustered by using the ‘FindNeighbors’ function with the first 15 principal components and the “FindClusters” function with 0.4 resolution. The clusters were visualized by the “RunUMAP” function. Marker genes of different clusters were found by ‘FindMarkers’ with parameters min.pct = 0.25 and logfc.threshold = 0.25). The known marker genes and high-expression genes of each cluster were used to annotate cell clusters. Four genes (ddx4, dazl, gdf9 and bmp15) were used as markers for oocytes [11,32,33].

## Figures and Tables

**Table 1:** Comparison of genome copy numbers of ZP genes in various species.

Species	ZP1	>ZP2	ZP3	ZP4	ZPAX	ZPD	Total Genes *	Chorion Thickness (µm)
Human	1	1	1	1	1p	0	5	-
Mouse	1	1	1	1p	0	0	4	-
Chicken	1	1	3	1	1	0	7	-
Red eared slider	2	3	8	4	0	0	17	-
Frog	0	1	4	4	1	1	11	-
Swordfish [25]	0	0	12	7	0	1	20	10–45
Tilapia [26]	0	0	12	6	3	1	22	5–6
Three spine stickleback [27]	0	0	39	7	1	1	48	8.5
Medaka [28]	0	0	15	5	5	1	26	10–15
Rainbow trout [27]	0	0	23 + 2p	14	0	1	40	14.7
Salmo salar [29]	0	2	44	9	1	4	60	30–38
Blue catfish	0	0	20 + 4p	13	1	1	39	-
Channel catfish	0	0	22 + 3p	19 + 2p	2	1	49	-
Common Carp [27]	1 + 1p	3	41	27	1	1	75	10–25
Zebrafish [30]	0	0	15	8	3	1	27	1.5–2.5

Letter “p” designates pseudogenes. ZP2, ZP3, and ZP4 are also referred to as ZPA, ZPC, and ZPB, respectively, in the literature. Teleost fish in general harbor high copies of ZP genes, significantly more than in higher vertebrates such as human, mouse, chicken, turtles, and frogs. Note that several species are allotetraploid or pseudo-autotetraploid (e.g., common carp and Atlantic salmon), which explains their relatively higher number of ZP genes. The rows for blue catfish and channel catfish are highlighted in light green. * Total genes included the number of pseudogenes.

**Table 4:** Primers for RT-PCR, along with annealing temperatures and expected PCR product sizes.

Gene Name	Forward Primer	Reverse Primer	Annealing Temperature	PCR Product Size
Beta-glucuronidase (gusb)	AAGATGGCCGATGTGTATCC	TATCCTGGGTTCTGGTGCAT	58.5 °C	103 bp
ZPAX1 (LOC128601446)	AGGCTTTCAGCATCGAGGTC	GCATAGAGGTCAGCCGAGAG	59 °C	293 bp
ZPD (LOC128611991)	AGTGCGTCTACCCGTTCATC	TGGGTACGCCTCCATAGACA	58 °C	156 bp
ZP3 (LOC128602850)	CAGCAGTAGCTGTGGTTTGAG	TGCTTGCACACTGCCCATTAC	60 °C	212 bp
ZP4 (LOC128621078)	CCACACACCCCTTTCCTAGTC	ATGCCGGTTAATGGCGTGT	58 °C	132 bp

## Data Availability

All RNA-Seq datasets have already been deposited in the SRA database of the NCBI. snRNA-Seq data have recently been deposited in the SRA database of the NCBI.

## Supplementary Materials

The following supporting information can be downloaded at: https://www.mdpi.com/article/10.3390/ijms27209100/s1.
